# Relative abundance of heavy metal resistance genes of three drinking water treatment plants unveiled through shotgun metagenomics

**DOI:** 10.3389/fmicb.2026.1822428

**Published:** 2026-04-21

**Authors:** Memory Tekere, Chimdi Mang Kalu

**Affiliations:** Department of Environmental Science, College of Agriculture and Environmental Sciences, University of South Africa, Florida, South Africa

**Keywords:** disinfection, drinking water, final treated water, heavy metal, raw water sources, resistance genes, shotgun metagenomics

## Abstract

**Introduction:**

The occurrence and abundance of heavy metal resistance genes (HMRGs) in drinking water treatment plants (DWTPs) and the stages at which they occur are a global challenge due to the risk of consuming contaminated water.

**Methods:**

The present study identified HMRGs associated with raw water sources, treatment stages (disinfection and filtration), final treated water, and produced sludge in three DWTPs across three provinces (Gauteng, Limpopo, and Mpumalanga) in South Africa, using a shotgun metagenomic approach.

**Results:**

In total, five classes of heavy metals (copper, arsenic, mercury, chromate, silver) and 50 resistance genes were identified across the three DWTPs. Most of the genes were obtained from the disinfection stages of the DWTPs.

**Discussion:**

This genomic dataset provides valuable information on the impact of disinfection stages on the relative abundance of HMRGs in drinking water treatment processes. Additionally, the transfer of genes into the final treated water consumed by the populace is a significant human health concern.

## 1 Introduction

Heavy metals, known for their low molecular weight, can cause environmental and human health problems at certain concentrations (Xavier et al., 2019). Their accumulation in the environment is mainly due to anthropogenic activities, which are difficult to reverse. Rivers, which form the source of raw water for the drinking water treatment plants (DWTPs), are faced with the challenge of heavy metals pollution due to illegal domestic sewage disposal, industrial activities that generate wastewater containing heavy metals, metals dragged by rains into the rivers, and discharge of poorly treated water from the wastewater treatment plants (Zampieri et al., 2016; Xu et al., 2016).

The accumulation of these metals in rivers and other aquatic ecosystems alters microbial composition and activity, causing some microorganisms to develop strong resistance to heavy metals (Sharma, 2012; Gupta and Diwan, 2017). This resistance to heavy metals by microorganisms could be linked to genetic alterations, leading to the formation of heavy metal resistance genes (HMRGs) that enable them to survive in the environment (Das et al., 2016). Studies have reported HMRGs such as *cadB* (cadmium), *chrA* (chromium), *pbrA* (lead), *MerA* (mercury), and *NiCoT* (nickel) in bacteria and their involvement in the transport of transition metals in water treatment processes (Janssen et al., 2010; Das et al., 2016).

DWTPs are responsible for producing good quality drinking water for human consumption; however, their effectiveness is linked to a variety of factors that include the quality of the raw water source, treatment processes and reagents used (Santana et al., 2014; Mensah-Akutteh et al., 2022; Pang et al., 2025). The treatment stages in DWTPs play a crucial role in the removal of microbial contaminants, antibiotic resistance genes, HMRGs, and other contaminants present in the raw water sources (Moreira et al., 2025). The abundance of bacteria possessing HMRGs, as well as the already released genes in the raw water source possess serious challenges to treat plants. Studies have shown that different treatment stages, especially in wastewater treatment plants, play a vital role in the removal of HMRGs (Azmi, 2025; Liu et al., 2025; Shukla et al., 2025). However, limited studies have been conducted using a shotgun metagenomic approach to reveal the abundance of HMRGs in stages of DWTPs in South Africa. Hence, this current study aimed at unveiling the abundance of HMRGs present in raw water sources, treatment stages, final treated water, and produced sludge in three DWTPs in three different provinces in South Africa using a shotgun metagenomic approach. The presence of heavy HMRGs in the final treated water and produced sludge may have a potential impact on public health.

## 2 Materials and methods

### 2.1 Selected study sites

Three different Drinking Water Treatment Plants (DWTPs) from three Provinces in South Africa were chosen for this study because the sites serve large populations, especially in urban and rural areas, and provide drinking water. The DWTPs’ names are coded as A (From Gauteng Province), B (from Limpopo Province), and C (from Mpumalanga Province) for confidentiality. The chosen study sites represent a large population in South Africa, especially in urban and rural areas where they provide drinking water (DWS, 2022). In Gauteng, Limpopo, and Mpumalanga provinces, the primary raw water sources are the Vaal River and Crocodile River catchments, Luvuvhu River catchment, and Upper Olifants River catchment, respectively, where many anthropogenic activities such as urbanisation, agriculture and industries occur along the river (Pollard et al., 2018; DWS, 2022).

### 2.2 Sampling procedure

Replicate samples were taken from raw water sources, treatment stages (disinfection stage and filtration stage), produced sludge, and the final treated water. Sterile 1,000 mL bottles were used to collect water samples from the raw water sources (from the river catchments of the three plants), the treatment plants (disinfection stage, filtration stage), produced sludge, and final treated water, and were transported at 4 °C to the laboratory for analysis within 3 to 6 h after collection. The triplicate samples represent independent random sampling events.

A total of 45 samples (5 sampling points × 3 Plants (A, B, and C) × 3 replicates on the sampling days per sampling points x 1 season) from all the sampling points. Samples were collected during the Spring of 2022 (in South Africa, the Spring falls within September, October, and November). To prevent external contamination of water samples collected from raw water sources, treatment stages (disinfection stage and filtration stage), produced sludge, and the final treated water in this study, strictly controlled procedures were employed, including the use of pre-sterilized or acid-washed sampling containers, disinfection and flushing of sampling points, and the use of clean personal protective equipment to avoid operator-induced contamination. Separate sampling tools were designated for each treatment stage, and containers were sealed immediately after collection to minimize exposure.

### 2.3 DNA extraction

One hundred millilitres of raw water and 1 L of water samples from the disinfection stage, filtration stage, and final treated water were filtered through a 0.22-μm polycarbonate membrane (purchased from Promolab Pty Ltd. T/A Separations, RSA). The risk of microbial contamination of the polycarbonate membrane filters was eliminated by using manufacturer-sterilized filters and handling them exclusively with sterile forceps inside a laminar-flow cabinet, along with autoclaved or single-use sterile filtration components. Additionally, filtration blanks were processed in parallel to verify sterility and ensure that any detectable DNA originated solely from the collected water samples. The filter papers with the collected microbial biomass were cut into small pieces and used for DNA extraction. Furthermore, 0.5 g of the produced sludge slurry, which contains the microbial biomass, was used for DNA extraction. DNA extraction was done (in the accredited microbiology and molecular biology laboratory in the University of South Africa) using MN-NucleoSpin DNA extraction kits (Promolab Pty Ltd. T/A Separations, RSA) following the manufacturer’s instructions.

### 2.4 DNA quantification and library preparations

DNA was extracted from three independent sample replicates, and equal quantities (5 ng each) of the resulting environmental DNA extracts were combined to generate a representative composite sample used for further analysis. DNA inhibitors were removed by applying extraction protocols that included inhibitor-removal chemistries, such as specialized binding resins, purification columns, and optimized washing buffers, capable of eliminating humic substances, metals, and residual disinfectants. For highly inhibitory matrices like disinfection and final sludge samples, additional clean-up steps (e.g., secondary silica-based purification or enhanced washing cycles) were used to ensure recovery of inhibitor-free DNA suitable for downstream molecular analyses. The DNA was quantified using a NanoDrop spectrophotometer (Thermo Fisher Scientific, United States), and the quality was checked on a 1.5% agarose gel. An obtained DNA with A260:A280 ratios between 1.8 and 2.0 and DNA concentrations of 20–150 ng/μL (according to internationally established standards for high-quality DNA, as recommended in the Thermo Fisher NanoDrop Spectrophotometer Guidelines and standard Illumina library preparation protocols) was sent to the Agricultural Research Council (ARC) Biotechnology Platform for shotgun metagenomic sequencing. The MGI DNBSEQ-G400 sequencing instrument was used by the ARC platform. The metagenomic shotgun data library was prepared using MGIEasy Universal DNA Library Prep Set V1.0 (MGI Tech Co., China). The quality control of the library was done using a Qubit® fluorometer (Life Technologies, Carlsbad, CA, United States), with the sequencing depth calculated to achieve five million reads. The DNA nanoball (DNB) was created by combining the pooled and circularized library, which was then loaded into a PE150 flow cell for sequencing in MGI DNBSEQ-G400 (MGI Tech Co., China) (2 × 150 bp).

### 2.5 Bioinformatics and visualization of sequencing data

Quality assessment of raw metagenomic shotgun sequencing (MSS) reads was performed using FastQC v1.0.0 (Andrews, 2010). Low-quality bases, ambiguous nucleotides, and sequencing adapters were removed using TrimGalore v0.6.5 (Krueger, 2015), applying a minimum average quality score threshold of Q15. To filter out human-derived reads, Kneaddata v0.11.0 was employed (Huttenhower Lab, 2018). WGSA2.2 pipeline implemented in Nephele (v2.2.8) (Weber et al., 2018) was used to curate the raw MSS comprising the sequences under default parameters. HMRGs were identified using AMRFinderPlus (v4.2.7) (Feldgarden et al., 2019), in the WGSA2.2 pipeline implemented in Nephele (v2.2.8) using default parameters to annotate high-confidence HMRGs determinants from the assembled metagenomic sequences. Stacked bar charts were generated from the identified classes of heavy metal resistance genes using the Paleontological Statistics Software Package Version 4 (PAST 4) (Hammer and Harper, 2001).

### 2.6 Statistical analysis

The three provinces in this study are dissimilar in demographic, industrial, rural–urban balance, or geological characteristics. To account for this inherent variability, the data were log_10_ normalized and statistical analysis was done on site-specific heavy metal metagenomes representing each province, ensuring that differences arising from urban runoff, industrial effluents, or natural mineral inputs are statistically adjusted. Heavy metal metagenomes were analyzed using one-way analysis of variance (ANOVA) with three replicates (*n* = 3) per treatment. Statistical analysis was performed using Statistica version 12 (StatSoft Inc., Tulsa, OK, United States). Mean separation was conducted using Duncan’s multiple range test at 95% significance level.

## 3 Results

### 3.1 Treatment plant a

**Figure 1:**
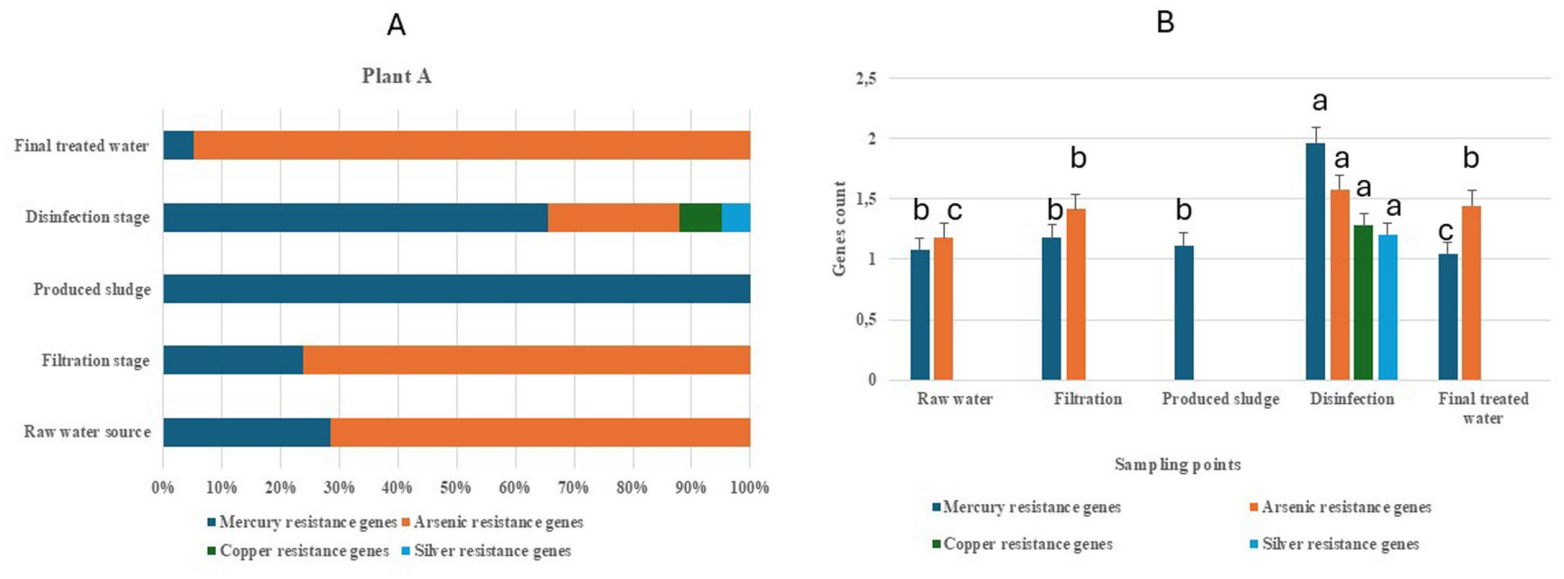
Heavy metal resistance genes associated with raw water sources, the disinfection stage, the filtration stage, final treated water, and the produced sludge from drinking water treatment plant A in the Gauteng Province. **(A)** Relative abundance of log_10_-normalized genes count. **(B)** Mean separation of gene counts across the sampling points [sampling points with the same letter (a–c) are not significant at *p* < 0.05].

In this plant, four classes of heavy metal resistance genes (HMRGs) have been identified. Table 1 provides a summary of the individual identified HMRGs and their HMM description in Gauteng Province. Variations in the relative abundance of HMRG classes were observed across raw water sources, treatment stages (disinfection and filtration), final treated water, and produced sludge (Figure 1). The raw water source, filtration stage, and final treated water contained both mercury and arsenic genes, with arsenic accounting for about 72, 75, and 97% of the genes, respectively. The disinfection stage contained all four heavy metal genes (mercury, arsenic, copper, and silver), with mercury accounting for about 67% of the genes. Produced sludge contained only mercury resistance genes (Figure 1A). To provide further insight into the significant impact of the treatment stages on the HMRGs, a statistical analysis that provides means separation across the sampling points was done. The mean separation showed that there was a significant difference (at *p* < 0.05) across the sampling points (raw water source, disinfection stage, filtration stage, final treated water and produced sludge) for mercury and arsenic resistance genes, with the disinfection stage showing the highest value for both metals (Figure 1B).

**Table 1:** Identified heavy metal resistance genes and the HMM description in treatment plant A (Gauteng Province).

Genes	Classes	HMM description
*arsN1*	Arsenic	Arsinothricin resistance N-acetyltransferase ArsN1 family B
*arsC*	Arsenic	Glutaredoxin-dependent arsenate reductase
*arsD*	Arsenic	Arsenite efflux transporter metallochaperone ArsD
*arsN2*	Arsenic	Arsenic resistance N-acetyltransferase ArsN2
*merP*	Mercury	Mercury resistance system periplasmic binding protein MerP
*merF*	Mercury	Mercury resistance system transport protein MerF
*merC*	Mercury	Organomercurial transporter MerC
*merA*	Mercury	Mercury (II) reductase
*merB*	Mercury	Organomercurial lyase MerB
*merD*	Mercury	Mercury resistance co-regulator MerD
*merE*	Mercury	Broad-spectrum mercury transporter MerE
*merG*	Mercury	Phenylmercury resistance protein MerG
*merR*	Mercury	Mercury resistance transcriptional regulator MerR
*merT*	Mercury	Not provided
*copS*	Copper	Not provided
*copR*	Copper	Not provided
*silS*	Silver	Not provided
*silA*	Silver	Not provided
*silB*	Silver	Not provided
*silF*	Silver	Not provided
*silC*	Silver	Not provided
*silR*	Silver	Not provided

### 3.2 Treatment plant B

**Figure 2:**
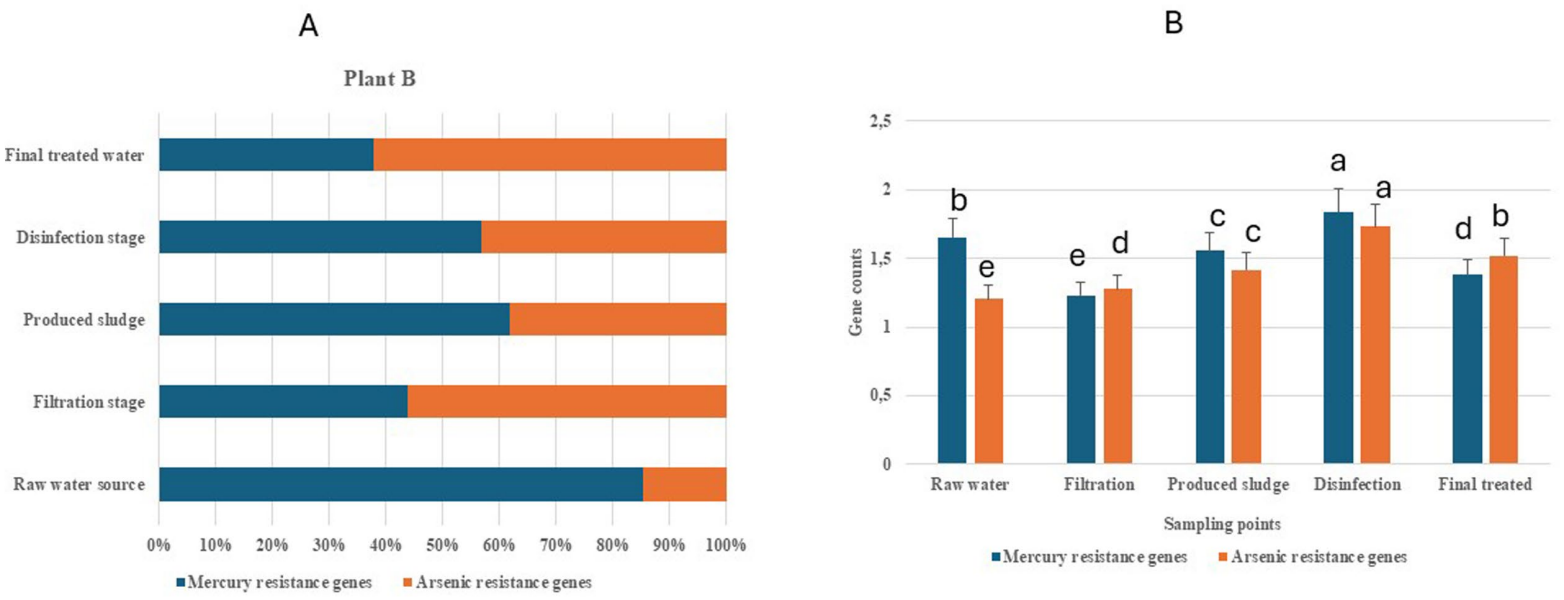
Heavy metal resistance genes associated with raw water sources, the disinfection stage, the filtration stage, final treated water, and the produced sludge from drinking water treatment plant B in the Limpopo Province. **(A)** Relative abundance of log_10_-normalized genes count. **(B)** Mean separation of gene counts across the sampling points [sampling points with the same letter (a–e) are not significant at *p* < 0.05].

Two classes of HMRGs (mercury and arsenic) were recorded for this plant. Table 2 provides a summary of the individual identified HMRGs and their HMM description in Limpopo Province. Variations in the relative abundance of the classes of HMRGs were observed across the raw water sources, treatment stages (disinfection and filtration stages), final treated water, and produced sludge, with the raw water sources recording about 86% of mercury and final treated water recording about 52% of arsenic metal (Figure 2A). In Figure 2B, the mean separation indicated a significant difference (at *p* < 0.05) across the sampling points (raw water source, disinfection stage, filtration stage, final treated water and produced sludge) for mercury and arsenic resistance genes, with the disinfection stage showing the highest value for both metals.

**Table 2:** Identified heavy metal resistance genes and the HMM description in treatment plant B (Limpopo Province).

Genes	Class	HMM description
*merE*	Mercury	Broad-spectrum mercury transporter MerE
*merD*	Mercury	Mercury resistance co-regulator MerD
*merA*	Mercury	Mercury (II) reductase
*merF*	Mercury	Mercury resistance system transport protein MerF
*merP*	Mercury	Mercury resistance system periplasmic binding protein MerP
*merT*	Mercury	Not provided
*merC*	Mercury	Organomercurial transporter MerC
*merG*	Mercury	Phenylmercury resistance protein MerG
*merR*	Mercury	Mercury resistance transcriptional regulator MerR
*arsN1*	Arsenic	Arsinothricin resistance N-acetyltransferase ArsN1 family B
*arsD*	Arsenic	Arsenite efflux transporter metallochaperone ArsD
*arsN2*	Arsenic	Arsenic resistance N-acetyltransferase ArsN2

### 3.3 Treatment plant C

**Figure 3:**
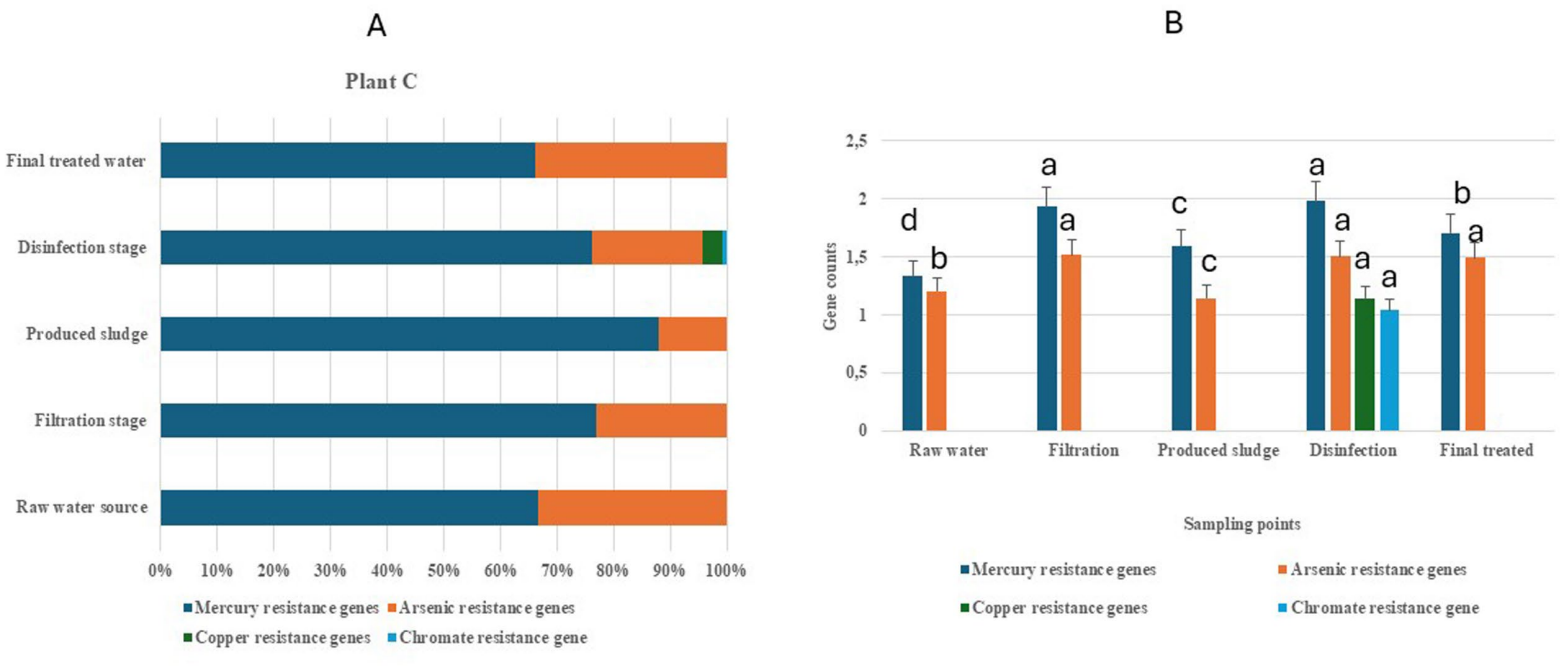
Heavy metal resistance genes associated with raw water sources, the disinfection stage, the filtration stage, final treated water, and the produced sludge from drinking water treatment plant C in the Mpumalanga Province. **(A)** Relative abundance of log_10_-normalized genes count. **(B)** Mean separation of gene counts across the sampling points [sampling points with the same letter (a–d) are not significant at *p* < 0.05].

Plant C recorded four classes of HMRGs (mercury, arsenic, copper, and chromate). Table 3 provides a summary of the individual identified HMRGs and their HMM description in Mpumalanga Province. Variations in the relative abundance of HMRG classes were observed across raw water sources, treatment stages (disinfection and filtration), final treated water, and produced sludge (Figure 3A). The raw water source, filtration stage, final treated water, and produced sludge contained both mercury and arsenic genes, with mercury accounting for about 65, 76, 65, and 88% of the genes, respectively. Disinfection stage contained all four heavy metal genes (mercury, arsenic, copper, and chromate), with mercury accounting for about 77% of the genes (Figure 3A). Significant difference (at *p* < 0.05) was observed for mercury and arsenic resistance genes across the sampling points (Figure 3B). The disinfection and filtration stage showed the highest value for mercury resistance genes. For arsenic resistance genes, the disinfection stage, filtration stage, and final treated water showed the highest values (Figure 3B).

**Table 3:** Identified heavy metal resistance genes and the HMM description in treatment plant C (Mpumalanga Province).

Genes	Class	HMM description
*merP*	Mercury	Mercury resistance system periplasmic binding protein MerP
*merA*	Mercury	Mercury (II) reductase
*merR*	Mercury	Mercury resistance transcriptional regulator MerR
*merT*	Mercury	Not provided
*merG*	Mercury	Phenylmercury resistance protein MerG
*merF*	Mercury	Mercury resistance system transport protein MerF
*merC*	Mercury	Organomercurial transporter MerC
*mere*	Mercury	Broad-spectrum mercury transporter MerE
*merD*	Mercury	Mercury resistance co-regulator MerD
*merB*	Mercury	Organomercurial lyase MerB
*arsN2*	Arsenic	Arsenic resistance N-acetyltransferase ArsN2
*arsD*	Arsenic	Arsenite efflux transporter metallochaperone ArsD
*arsN1*	Arsenic	Arsinothricin resistance N-acetyltransferase ArsN1 family B
*chrA*	Chromate	Not provided
*copR*	Copper	Not provided
*copS*	Copper	Not provided

## 4 Discussion

Treatment stages in DWTPs affect the water’s microbial ecology due to varying conditions (Zhao et al., 2025). Each stage of treatment has specific functions, and these functions determine the effectiveness and efficiency of the treatment in the production of quality drinking water (Ma et al., 2017). In this study, the analysis focused on the disinfection stage and filtration stage, whose functions are mainly focused on the reduction/removal of microbial contaminants, resistant genes, including HMRGs, from the water undergoing treatment (Dai et al., 2020; Nakazawa et al., 2021; Borrull et al., 2021; Zhao et al., 2025). Generally, our results showed that the disinfection stage of all the treatment plants contributed to the abundance of different classes of HMRGs when compared to the raw water sources. In addition, different treatment plants exhibited variations in HMRG classes, which could be attributed to anthropogenic activities around the raw water sources used by the treatment plants (Zampieri et al., 2016; Xu et al., 2016).

Differences in HMRG profiles across the raw water, filtration, and disinfection stages, the final treated water, and the produced sludge are consistent with the changing ecological conditions encountered throughout drinking water treatment (Jia et al., 2020). Raw water naturally reflects site-specific watershed inputs, industrial activities, and geochemical conditions, which can shape distinct resistome profiles, as demonstrated by Hemme et al. (2010), who showed that metal-rich environments select for unique resistance-gene communities driven by long-term contamination pressures. During treatment, filtration concentrates biomass and associated resistance determinants into sludge, a pattern also observed by Li et al. (2015), who reported marked differences in metal-resistance gene abundance between influent and sludge fractions due to biomass accumulation and plasmid-mediated gene transfer in treatment systems. According to Li et al. (2015), filtration may fail to fully remove heavy-metal resistance genes because eDNA and mobile genetic elements can pass through filter media. Disinfection introduces strong oxidative and chemical stress that favors metal-tolerant microorganisms, similar to findings by Gupta et al. (2018), who noted that heavy-metal resistance genes often persist or increase after treatment because resistant taxa are selectively retained. Additional metagenomic studies, including Enahoro (2025) and Naccache et al. (2025), further show that co-selection with antibiotic-resistance genes and enrichment of mobile genetic elements contribute to heterogeneity across treatment stages. These mechanisms collectively explain the stage-specific and plants-specific patterns of HMRGs observed in the present study.

Raw water sources are the starting point of the DWTPs, and they have different chemical and physical properties, which tend to determine the microbial constituent (Zhao et al., 2025). The nature and load of the contaminants could play a role in how the DWTPs’ stages perform to ensure good quality of drinking water (Feng et al., 2023). The disinfection stage, which aimed at the removal of microbial contaminants as well as the associated resistant genes present in the raw water sources, also has a challenge of releasing more resistant genes from the microbial communities (Tiwari et al., 2022). This aligns with the findings in this current study, where, in Plant A, all four classes of HMRGs (mercury, arsenic, copper, and silver) occurred, and in Plant C, where all four classes of HMRGs (mercury, arsenic, copper, and chromium) occurred when compared to the raw water sources.

The filtration stage is essential in the treatment process because of its role in the removal of microorganisms (Cescon and Jiang, 2020) and antibiotic-resistant genes (Gao et al., 2022). The role of the filtration stage in the removal of resistance genes was observed in this study, especially in Plant A and Plant C. In plant A, only HMRGs of mercury and arsenic were observed, indicating the removal of copper and silver during the stage. This was also evident in the final treated water. In Plant C, only HMRGs of mercury and arsenic were observed, indicating the removal of copper and chromium during the stage. This was also evident in the final treated water.

In the final treated water for all the plants, mercury resistance genes and arsenic resistance genes were dominant, with variations in the treatment plants. This is a great health concern as the final treated water is being consumed by the populace in that province. Genchi et al. (2017) highlighted that mercury exposure could lead to heart disease. Exposure to arsenic could lead to cancer, skin disorders, and cognitive impairment, among other conditions (Chen et al., 2021). Consumption of water with these resistance genes could expose humans to these diseases, as these genes can penetrate through the cell structures as well as bacteria that are normal flora to human beings and cause diseases (Ellwanger et al., 2025).

Heavy-metal exposure has been linked internationally to cancers, cardiovascular disease, neurological impairment, and skin disorders, and several South African studies highlight similar risks in provinces where this study was conducted. In Gauteng, Kootbodien et al. (2026) reported elevated arsenic and lead in soils around early learning centres, noting associated risks for childhood cognitive impairment, carcinogenesis, and skin lesions. In Limpopo, Maliehe et al. (2024) identified groundwater contamination with lead and vanadium, finding both non-carcinogenic and carcinogenic risks for residents, particularly through long-term exposure pathways. Additional work by Kapwata et al. (2020) in rural Limpopo showed significant arsenic contamination linked to abandoned mining areas, emphasizing risks for skin lesions, cardiovascular effects, and reduced IQ in exposed populations. In view of this, heavy-metal exposure (including the ones from final treated water) may pose real public-health risks within these provinces.

## 5 Conclusion

The production of potable water of good microbiological and aesthetic status is pertinent to the health of human beings who depend on the water for drinking, domestic activities, and other activities necessary for survival. The presence of contaminants and genes in the final treated water is a drawback to the roles of the DWTPs. In this study, the disinfection stage is the contributing factor to the abundance and diversity of HMRGs, and the filtration stage’s function in the removal of these genes was also minimal, as observed in the final treated water. This is a concern for the treatment plants. Studies are recommended in the development of real-time technology that can take into account the presence of these genes during the treatment stages and promote sustainable removal of these genes.

## Data Availability

All data analysis results from this study are included in the manuscript. All the raw datasets have been deposited at the NCBI database (https://www.ncbi.nlm.nih.gov/) sequence archive (SRA) as BioProject ID PRJNA1090776.
